# Loneliness and Social Functioning in Adolescent Peer Victimization

**DOI:** 10.3389/fpsyg.2021.664079

**Published:** 2021-07-01

**Authors:** Telma Sousa Almeida, Olivia Ribeiro, Miguel Freitas, Kenneth H. Rubin, António J. Santos

**Affiliations:** ^1^William James Center for Research, ISPA—Instituto Universitário, Lisbon, Portugal; ^2^Department of Human Development and Quantitative Methodology, University of Maryland, College Park, MD, United States

**Keywords:** adolescence, interpersonal adversity, peer victimization, aggressive behavior, anxious-withdrawal, loneliness

## Abstract

Interpersonal adversity such as peer victimization has been shown to have complex associations with other socio-emotional difficulties, particularly during adolescence. We used a multidimensional peer nomination measure on a sample of 440 (52% girls) 11- to 17-year-old (M = 13.14 years, SD = 1.26) Portuguese youths to identify three groups, classified by peers as (1) victimized adolescents who showed anxious withdrawn behaviors in the context of the peer group (*n* = 111), (2) victimized adolescents who did not exhibit anxious withdrawn behaviors (*n* = 104), and (3) non-victimized adolescents (*n* = 225). We compared these groups on their peer-reported social functioning and on their self-reported feelings of social and emotional loneliness (with peers and family). Anxiously withdrawn victims were viewed by peers as more excluded, less aggressive, less prosocial, and less popular than non-withdrawn victims and non-victims. Non-anxiously withdrawn victims were considered more excluded than non-victims, and more aggressive than both anxiously withdrawn victims and non-victims. Finally, anxiously withdrawn victims reported feeling less integrated and intimate with their peers than non-withdrawn victims and non-victims, which is indicative of greater feelings of social and emotional loneliness at school. Youths in the current study did not report feeling lonely in their family environment. Our findings thus provide further evidence that victimized youths constitute a heterogeneous group, which differ in the way they behave toward their peers and experience loneliness.

## Introduction

Youths are frequently targets of direct or indirect intentional aggressive behaviors by peers [World Health Organization (WHO), 2017]. More than 150 million youths (13-15 year-olds) worldwide report experiencing peer violence in and around school (UNICEF, 2019). Being victimized by peers has a major impact on development and well-being and has long-lasting consequences that can lead to short and long-term adjustment difficulties in multiple domains (e.g., Arseneault, 2018).

It is well-established that victimized youths constitute a heterogeneous group and researchers recognize the need to distinguish different subgroups among adolescents who are victimized by peers (e.g., Guedes et al., 2018). There are those who are aggressive and provocative and thus elicit victimization by peers (e.g., Hanish and Guerra, 2004; Rubin et al., 2006, 2015). Others, however, are withdrawn, submissive, physically and/or emotionally vulnerable, and consequently perceived by peers as passive, easy targets (e.g., Hanish and Guerra, 2004; Rubin et al., 2006, 2015).

Anxious-withdrawn youths are at high risk for victimization (Boivin and Hymel, 1997; Kochenderfer-Ladd, 2003; Hanish and Guerra, 2004; Boivin et al., 2010; Freitas et al., 2019). They may display submissive behaviors, which are often interpreted by peers as strange, irritating, and deviating from social norms and expectations for social interaction (Erath et al., 2008; Rubin et al., 2015). These shy, subservient behaviors can increase their vulnerability to peer victimization (Olweus, 1993). Furthermore, withdrawn, victimized children frequently become more withdrawn over time when victimization endures (Gazelle and Rudolph, 2004; Boivin et al., 2010; Wang et al., 2014). Longitudinal research has consistently demonstrated that anxious withdrawal is one of the strongest individual correlates of victimization, with withdrawal predicting later victimization, and victimization predicting later withdrawal (e.g., Salmivalli, 2001; Schwartz et al., 2001; Boivin et al., 2010; Wang et al., 2014).

Being victimized by peers thus influences youths' social behavior. Youths who are victims of peer aggression often avoid social interactions with those whom they fear or mistrust (Gazelle and Rudolph, 2004). On some occasions, they may also attribute maltreatment to characteristics they have (Graham et al., 2006; Perren et al., 2013) and, consequently, withdraw from peers due to feelings of embarrassment and negative self-perceptions (e.g., Troop-Gordon and Ladd, 2005). Prior research thus suggests that anxious withdrawal (individual vulnerability) and victimization (interpersonal adversity) interact in reciprocal and mutually exacerbating ways (Rubin et al., 2018).

Although current literature shows the importance of distinguishing between the constructs of victimization, rejection, and exclusion (Rubin et al., 2015), these social functioning dimensions seem, nevertheless, to be closely associated. Prior research shows that victimized youths experience more interpersonal adversity, including rejection by their peers, especially when they display hostile and aggressive behavior (Juvonen et al., 2003; Nansel et al., 2004; Veenstra et al., 2005; Leiner et al., 2014).

Also, Coie (1990) and Buhs and Ladd (2001) highlighted that rejected children are usually disliked and consequently maltreated by their peers. Once identified as targets for maltreatment, rejected children become even more marginalized or excluded by peers. Buhs et al. (2006) have shown that the construct of peer rejection was associated with distinct forms of peer maltreatment (i.e., abuse and exclusion) that endured over many school years and resulted in adverse school adjustment outcomes (e.g., school disengagement; decreased classroom participation). Being disliked and consequently rejected and excluded by peers may be a strong motivation for maltreatment (Buhs et al., 2006; Sentse et al., 2015).

Victimized children are perceived by peers and teachers as being less prosocial (Perren et al., 2013; Pouwels and Cillessen, 2013) and less popular in school than non-victimized peers (Juvonen et al., 2003; Veenstra et al., 2005). Particularly, this negative impact on one's social reputation seems to be greater among aggressive victims when compared with non-aggressive victims (Juvonen et al., 2003; Veenstra et al., 2005).

In a longitudinal study, Rudolph et al. (2014) found that both early and increasing victimization predicted heightened social helplessness and dampened prosocial behavior in sixth grade, following the transition to middle school.

Evidence gathered over the last decades has consistently indicated that youth victimization is associated with distress and emotional, psychological, social and academic adjustment problems (Nansel et al., 2001; Arseneault, 2018). Victims of peer maltreatment often develop internalizing difficulties such as anxiety, depression, self-blame, hopelessness, and helplessness (Olweus, 1995; Veenstra et al., 2005; Salmivalli and Peets, 2009).

Previous research has consistently shown that loneliness is a strong correlate of peer victimization across development and cultures and regardless of the measurements (i.e., self- vs. peer reports) used to investigate this relationship (e.g., Kochenderfer and Ladd, 1996; Boivin and Hymel, 1997; Graham et al., 2006; Card and Hodges, 2010; Woodhouse et al., 2012; Ribeiro et al., 2019).

Being victimized by peers, particularly when youths are explicitly disliked or rejected, appears to encourage anxiously-withdrawal behaviors, which in turn may impact on the opportunities youths have to interact within the peer group and develop significant relationships (Haynie et al., 2001; Schwartz et al., 2001; Veenstra et al., 2005; Leary et al., 2006). Thus, it is understandable that many victimized children (Kochenderfer and Ladd, 1996) and adolescents (Storch and Masia-Warner, 2004) report experiencing elevated levels of loneliness (Nansel et al., 2001; Liu et al., 2019). Furthermore, it has been argued that the perceived lack of control over peers' aggressive behavior may generate enhanced feelings of loneliness and unhappiness (Boulton and Underwood, 1992; Nansel et al., 2001, 2004).

Loneliness may be best understood as a multidimensional construct (e.g., Ribeiro et al., 2019). Drawing on Weiss's Social Needs Perspective 1973, it is imperative to distinguish between social and emotional forms of loneliness. Social loneliness (or integration), associated with the absence of engagement, consists in a lack of integration in social networks that could offer a sense of connection with others. Emotional loneliness (or intimacy), i.e., the absence of satisfactory close relationships, refers to the lack of intimacy that could provide a sense of share and trust. It is an affective state that is unrelated to the number of friendships formed (Ribeiro et al., 2019).

Notwithstanding, family and peer relationships are significant developmental contexts for youth and each one offers unique provisions (Weiss, 1973). However, few researchers have explored the distinction in quantitative and qualitative features of social relationships (i.e., intimacy and integration) in such two relational contexts.

The few studies exploring the impact of the family environment on victimized children's psychosocial adjustment (Gerard and Buehler, 1999; Johnson et al., 2001; Lucia and Breslau, 2006; Guedes et al., 2018) suggest that a lack of open communication, affection, support and secure attachment to parents may not only increase the risk for peer victimization at school, but also affect youths' psychological adjustment, exacerbating, for example, feelings of loneliness (Johnson et al., 2001). More studies are needed to understand how peer victimization reflects on youths' psychological adjustment within the family system.

Most peer victimization literature has used self-report measures (Haynie et al., 2001; Nansel et al., 2004; Unnever, 2005; Leiner et al., 2014) to identify different groups of victimized adolescents. Only a few studies have included peer nomination measures (Juvonen et al., 2003; Veenstra et al., 2005; Guedes et al., 2018), but these have not assessed the multiple dimensions of youths' positive and negative social behaviors, which are typical of the vast majority of adolescents. New studies are needed to provide a better understanding of the broader social behavior of withdrawn and non- withdrawn victims, using multidimensional peer nomination measures. Such assessments are advantageous because they represent the perspectives of many observers. Furthermore, peers are privileged informants about the interactions, relationships, and integration within the group of those being evaluated (Correia et al., 2014a; Rubin et al., 2015; Bukowski et al., 2018).

### The Present Study

In the current study, we examined positive and negative dimensions of social functioning (peer victimization, anxious-withdrawal, peer exclusion, aggression, prosocial behavior, and popularity/sociability) in a sample of Portuguese adolescents, classified by peers as anxious-withdrawn victims, non-withdrawn victims, and non-victims.

Furthermore, we examined two different forms of perceived loneliness – social (lack of integration) and emotional (lack of intimacy) – experienced by anxious-withdrawn victims, non-withdrawn victims, and non-victims in their two major contexts of socialization (i.e., peer group and family). For each study aim, sex and age were controlled.

Informed by previous research, we expected that anxious-withdrawn victims would display lower levels of aggression than the other groups of adolescents. We also predicted that anxious-withdrawn and non-withdrawn victims would display higher levels of peer exclusion and lower levels of prosocial behavior and popularity/sociability than non-victims; but anxious-withdrawn victims would display lower scores for these dimensions than non-withdrawn victims. Finally, both anxious-withdrawn and non-withdrawn victims would report feeling lonelier, when compared to non-victims, in both contexts of socialization.

## Methods

### Participants

Participants are part of a larger longitudinal research project (PTDC/PSI-PDE/098257/2008) and were recruited from three Portuguese public junior high schools in lower middle-class neighborhoods in Metropolitan Lisbon. The present study included 440 (*n* = 230 girls) 11- to 17-year-old adolescents (M = 13.14 years, SD = 1.26). Most participants (*n* = 360) were in the seventh grade, 63 were in the eighth grade, and 17 were in the ninth grade.

### Procedure

This study was approved by ISPA-Instituto Universitário's Ethical Committee. We obtained permission to collect the data from school boards, as well as written informed consent and assent from all participating families and adolescents. Personal data collection and processing followed the Declaration of Helsinki, APA guidelines, and the European General Data Protection Regulation, ensuring the privacy and confidentiality of participants' information.

Two researchers administered the questionnaires to students in group sessions lasting 45 min. Non-participating adolescents remained in the classroom, completing tasks assigned by their teacher.

### Measures

#### Extended Class Play

The ECP (Burgess et al., 2006; Portuguese version: Correia et al., 2014a) has 37 items and assesses peers' evaluations of the participants' social functioning in six dimensions: peer victimization, anxious-withdrawal, peer exclusion, aggression, prosocial behavior, and popularity/sociability (Cronbach's α were 0.80, 0.84, 0.81, 0.85, 0.69, and 0.73, respectively). Participants were instructed to pretend to be the directors of an imaginary class play and to nominate, among their participating classmates, one girl and one boy who would perform better in each of the 37 positive and negative roles. We only considered same-sex nominations to eliminate possible sex-stereotyping (Zeller et al., 2003). We summed and standardized all item scores within sex and classroom to adjust for classroom size differences (Cillessen and Bukowski, 2018). Finally, we calculated each dimension's mean composite by averaging the corresponding standardized individual item scores.

#### Relational Provision Loneliness Questionnaire

The RPLQ (Hayden-Thomson, 1989; Portuguese version: Ribeiro et al., 2019) has 28 self-report items and assesses subjective feelings of loneliness through two aspects of social satisfaction (group integration and personal intimacy), experienced in two different social contexts—within the peer group and family. This multidimensional measure comprises the following four subscales: peer-group integration, peer-personal intimacy, family-group integration, and family-personal intimacy (Cronbach's α were 0.84, 0.88, 0.90 and 0.92, respectively). Adolescents rated how true each statement was for them on a five-point Likert scale ranging from 1 (not at all true) to 5 (always true). Scores on each subscale ranged from 7 to 35. To estimate adolescents' perceived loneliness, we reverse coded all item scores and calculated the mean scores within each specific relationship (peers and family) and type of relational benefit (integration and intimacy). Higher scores in each subscale indicated higher levels of loneliness.

### Identification of Risk and Comparison Groups

We used the ECP scores to classify adolescents in three groups, following previously described procedures (e.g., Ladd and Burgess, 1999; Burgess et al., 2006; Rubin et al., 2006; Guedes et al., 2018): victims who were anxiously withdrawn (*n* = 111), victims who were not anxiously withdrawn (*n* = 104) and non-victims (*n* = 225). The group of anxiously withdrawn victims (AWV) comprised participants whose standardized scores in the dimensions of peer victimization and anxious withdrawal were in the top 33%. The group of non-anxiously withdrawn victims (nAWV) comprised participants whose peer victimization standardized scores were in the top 33% and whose anxious withdrawal standardized scores were below the respective median. The group of non-victims (NV) comprised participants who displayed scores below the respective medians in both dimensions of peer victimization and anxious withdrawal.

### Analysis Plan

We computed correlations between all studied variables (Table 1), descriptive statistics for sample characterization and examined the social functioning and perceived feelings of loneliness of the three groups of adolescents using the multivariate analyses of covariance (MANCOVA). Group was entered as the between-subjects variable, age and sex as covariates, and each social functioning dimension (peer victimization, anxious withdrawal, peer exclusion, aggression, prosocial behavior and popularity/sociability) and loneliness subscale (peer-group integration, peer-personal intimacy, family-group integration, and family-personal intimacy) as the dependent variables.

**Table 1:** Correlations between Study Variables.

		**2**	**3**	**4**	**5**	**6**	**7**	**8**	**9**	**10**
	**Social Functioning**									
1	Anxious Withdrawal	0.50^*^	0.67^*^	−0.26^*^	−0.14^*^	−0.26^*^	0.22^*^	0.13^*^	−0.07	−0.05
2	Peer Victimization		0.70^*^	0.03	−0.18^*^	−0.33^*^	0.20^*^	0.11^*^	−0.05	−0.03
3	Peer Exclusion			−0.03	−0.23^*^	−0.33^*^	0.26^*^	0.16^*^	−0.07	−0.06
4	Aggression				−0.16^*^	0.12^*^	−0.04	0.04	0.01	0.03
5	Prosocial Behavior					0.46^*^	−0.15^*^	−0.17^*^	−0.05	−0.10^*^
6	Popularity						−0.18^*^	−0.17^*^	−0.01	−0.05
	**Loneliness**									
7	Peer-Group Integration							0.52^*^	0.36^*^	0.26^*^
8	Peer-Personal Intimacy								0.28^*^	0.31^*^
9	Family-Group Integration									0.65^*^
10	Family-Personal Intimacy									

* *p < 0.05*.

Pillai's trace criterion (V) was selected as the multivariate test to assess the statistical significance of the group effect, due to its robustness with unequal sample sizes (Tabachnick and Fidell, 2007). When a significant multivariate effect was identified, we conducted univariate analyses of covariance (ANCOVA), followed by pairwise comparisons with Bonferroni corrections. All statistical comparisons were two-tailed, using *p* < 0.05 as the level of significance. Effect sizes are indicated by partial eta-squared ($\eta_{p}^{2}$).

## Results

### Social Functioning and Reputation

The top six rows of Table 2 show the means and standard deviations for all dimensions of social functioning for each group. After controlling for sex and age, there was a significant multivariate effect of group on social functioning, V = 1.24, *F*_(12,852)_ = 116.20, *p* < 0.001, $\eta_{p}^{2}$ = 0.62, π = 1.00. There was a significant multivariate effect of age, V = 0.04, *F*_(6,425)_ = 2.70, *p* = 0.014, $\eta_{\text{p}}^{2}$ = 0.04, π = 0.87, but no effect of sex, V = 0.01, *F*_(6,425)_ = 0.44, *p* = 0.853, $\eta_{\text{p}}^{2}$ = 0.01, π = 0.18.

**Table 2:** Means and standard deviations for all dimensions of social functioning and perceived feelings of loneliness, by group.

	**Group**						
	**Anxiously withdrawn victims**		**Non-anxiously withdrawn victims**		**Non-victims**		**Group effects**
	**M**	**SD**	**M**	**SD**	**M**	**SD**	
**Social Functioning**							
Anxious Withdrawal	1.13	0.44	−0.54	0.43	−0.53	0.44	AWV>N-AWV^*^
Peer Victimization	0.98	0.55	0.79	0.55	−0.54	0.55	AWV=N-AWV>N-V^*^
Peer Exclusion	0.95	0.66	0.01	0.66	−0.42	0.66	AWV>N-AWV>N-V^*^
Aggression	−0.18	0.65	0.36	0.65	0.06	0.65	AWV<N-V<N-AWV^*^
Prosocial Behavior	−0.24	0.62	−0.07	0.62	0.03	0.62	AWV<N-V^*^
Popularity	−0.31	0.67	−0.08	0.67	0.22	0.67	AWV<N-AWV<N-V^*^
**Loneliness**							
Peer-Group Integration	2.28	0.70	2.01	0.70	1.95	0.70	AWV<N-AWV=N-V^*^
Peer-Personal Intimacy	1.92	0.74	1.75	0.75	1.65	0.75	AWV<N-V^*^
Family-Group Integration	1.55	0.80	1.6	0.80	1.66	0.80	
Family-Personal Intimacy	1.59	0.86	1.68	0.86	1.68	0.86	

*AWV, Anxiously withdrawn victims; N-WV, Non-anxiously withdrawn victims; N-V,Non-victims*;

* *p < 0.05*.

Univariate analyses of covariance supported the presence of statistically significant group differences in all social functioning dimensions. Specifically, there was a significant effect of group on peer victimization, *F*_(2,430)_ = 365.71, *p* < 0.001, $\eta_{\text{p}}^{2}$ = 0.63, π = 1.00; anxious withdrawal, *F*_(2,430)_ = 598.32, *p* < 0.001, $\eta_{\text{p}}^{2}$ = 0.74, π = 1.00; peer exclusion, *F*_(2,430)_ = 156.78, *p* < 0.001, $\eta_{\text{p}}^{2}$ = 0.42, π = 1.00; aggression, *F*_(2,430)_ = 18.04, *p* < 0.001, $\eta_{\text{p}}^{2}$ = 0.08, π = 1.00; prosocial behavior, *F*_(2,430)_ = 6.85, *p* = 0.001, $\eta_{\text{p}}^{2}$ = 0.03, π = 0.92; and popularity/sociability dimensions, *F*_(2,430)_ = 23.78, *p* < 0.001, $\eta_{\text{p}}^{2}$ = 0.10, π = 1.00.

Pairwise comparisons with Bonferroni correction showed that both anxiously withdrawn and non-anxiously withdrawn victims displayed higher scores in the dimension of peer victimization compared to non-victims. These comparisons also showed that anxiously withdrawn victims exhibited higher scores in the dimension of anxious withdrawal compared to non-anxiously withdrawn victims. These results confirm the correct allocation of adolescents to the different groups.

Moreover, both anxiously withdrawn and non-withdrawn victims showed higher scores in the peer exclusion dimension than non-victims, although anxiously withdrawn victims had higher scores in this dimension than non-withdrawn victims. Non-anxiously withdrawn victims had higher scores for the aggression dimension compared to both anxiously withdrawn victims and non-victims. Non-victims displayed higher scores in the dimension of aggression than anxiously withdrawn victims. In addition, anxiously withdrawn victims evidenced lower scores for the prosocial behavior dimension compared to non-victims. Finally, both anxiously withdrawn and non-anxiously withdrawn victims showed lower scores in the popularity/sociability dimension than non-victims. Non-anxiously withdrawn victims had higher scores in this dimension than anxiously withdrawn victims.

### Subjective Feelings of Loneliness

The bottom four rows of Table 1 show means and standard deviations for each specific relationship (peers and family) and type of relational benefit (integration and intimacy), for each group. After controlling for sex and age, there was a significant multivariate effect of group on loneliness, V = 0.06, *F*_(8,866)_ = 3.44, *p* = 0.001, $\eta_{\text{p}}^{2}$ = 0.03, π = 0.98. There was a significant multivariate effect of sex, V = 0.12, *F*_(4,432)_ = 14.48, *p* < 0.001, $\eta_{\text{p}}^{2}$ = 0.12, π = 1.00, but no effect of age, V = 0.02, *F*_(4,432)_ = 1.76, *p* = 0.137, $\eta_{\text{p}}^{2}$ = 0.02, π = 0.54. Univariate analyses of covariance evidenced the presence of statistically significant group differences in peer-group integration, *F*_(2,435)_ = 8.19, *p* < 0.001, $\eta_{\text{p}}^{2}$ = 0.04, π = 0.96, and peer-personal intimacy, *F*_(2,435)_ = 4.60, *p* = 0.011, $\eta_{\text{p}}^{2}$ = 0.02, π = 0.78, but not in family-group integration, *F*_(2,435)_ = 0.63, *p* = 0.534, $\eta_{\text{p}}^{2}$ = 0.00, π = 0.16, or family-personal intimacy, *F*_(2,435)_ = 0.43, *p* = 0.653, $\eta_{\text{p}}^{2}$ = 0.00, π = 0.12.

Pairwise comparisons with Bonferroni correction showed that anxiously withdrawn victims reported feeling less integrated in the peer group than either non-anxiously withdrawn victims or non-victims, who did not differ from each other. They also reported feeling less intimate in their relationships within the peer group than non-victims.

## Discussion

This study represents the first attempt to explore social functioning and perceived social and emotional loneliness across different types of Portuguese victimized youths. We used a multidimensional peer-nomination measure, including both positive and negative dimensions of social behavior, and a self-report measure that focused on perceived feelings of loneliness in the two major contexts of youths' socialization. Our findings corroborated previous research and added to the current knowledge on peer victimization by demonstrating that anxiously withdrawn victims of peer maltreatment differ from victimized youths who are not anxiously withdrawn and those who are not victimized in the way they behaved toward their peers and experienced loneliness.

Our results demonstrate that anxiously withdrawn and non-withdrawn victims were more excluded by peers than non-victims. These findings seem to reinforce the notion that, although distinct, the constructs of victimization and exclusion are closely intertwined. In line with previous research (Coie, 1990; Buhs and Ladd, 2001; Buhs et al., 2006), our findings also suggest that victimized youths were viewed by classmates as actively marginalized or excluded by peers. Anxiously withdrawn victims were, however, perceived by classmates as being even more excluded by the peer group than non-withdrawn victims. These results are in line with previous research (Gazelle and Rudolph, 2004; Hanish and Guerra, 2004; Boivin et al., 2010) suggesting that anxiously withdrawn adolescents are usually disliked and perceived by peers as less socially competent, which in turn is associated with several forms of peer maltreatment, such as exclusion and physical, verbal, or relational victimization (Coie, 1990; Buhs and Ladd, 2001; Buhs et al., 2006; Sentse et al., 2015; Liu et al., 2019).

According to peers, non-anxious withdrawn victims displayed more aggressive behaviors in school than anxious withdrawn victims and non-victims. Our findings thus provided further evidence that victimized youths constitute a heterogeneous group and that victimization and aggression are not mutually exclusive concepts (e.g., Haynie et al., 2001; Rudolph et al., 2014).

Prior research indicates that there is a subgroup of victims who are prone to aggressive and provocative behaviors (Haynie et al., 2001; Olweus, 2001; Schwartz et al., 2001; Veenstra et al., 2005; Rudolph et al., 2014). These have been referred to in the literature as aggressive victims (Schwartz et al., 2001; Hanish and Guerra, 2004; Guedes et al., 2018), provocative victims (Olweus, 2001), and bully/victims (Haynie et al., 2001; Veenstra et al., 2005). Our study supports this view, showing that some peer victimized youths may engage in proactive and/or reactive aggression and thus be identified by peers as violent and hostile. Research suggests that some aggressive victims exhibit impulsive and proactive hostile behaviors, as an attempt to re-establish their status in the peer group (Haynie et al., 2001; Unnever, 2005), and may consequently provoke peer victimization (Olweus, 2001). Others simply engage in reactive aggression; that is, when provoked or maltreated by peers, they respond by retaliating and being hostile toward bullies (Olweus, 2001; Unnever, 2005) and may thus be perceived by their peers as being violent, as shown by the present results.

Furthermore, our results illustrated that anxiously withdrawn victims were considered significantly less aggressive by their peers than both victimized adolescents who did not exhibit anxious withdrawn behaviors and non-victimized adolescents. In line with previous findings (e.g., Haynie et al., 2001; Schwartz et al., 2001; Veenstra et al., 2005; Leary et al., 2006; Correia et al., 2014b), anxiously withdrawn victims represented a subgroup of youths who responded passively and non-aggressively to peer victimization. By avoiding or withdrawing from social interaction and displaying shy and submissive behaviors, anxiously withdrawn adolescents may be perceived as vulnerable targets for peer maltreatment, because they are unlikely to retaliate against aggressors (Rubin et al., 2015).

Several researchers suggest that youths identified as victims, particularly those who are withdrawn or submissive, exhibit fewer prosocial behaviors than non-victimized peers (e.g., Schwartz, 2000; Perren et al., 2013; Pouwels and Cillessen, 2013; Rudolph et al., 2014). They are also considered less popular than non-victimized peers (Juvonen et al., 2003; Veenstra et al., 2005). Similarly, we found that anxiously withdrawn victims were viewed as less prosocial than non-victims by their classmates, and, as expected, both anxiously withdrawn and non-anxiously withdrawn victims were characterized by peers as being less popular than non-victims. Altogether, these findings might suggest that both groups of victimized youths appear to display a diminished repertoire of social skills (either by being provocative or, conversely, shy and timid), which deviates from social norms and expectations. Such poorer interactive styles are consistent with the notions of moving against and away from the peer group (Gazelle and Rudolph, 2004) and, therefore, seem to associate with lower social status.

We found that anxiously withdrawn victims reported feeling less integrated in their peer group than either non-anxiously withdrawn victims or non-victims. They also reported feeling less intimate in their relationships within the peer group, which suggests that victimized adolescents in the current study, particularly those who were anxiously withdrawn, felt lonelier at school than non-victimized peers. These results are consistent with previous findings showing a strong link between peer victimization and internalizing difficulties such as intense feelings of loneliness (Boivin and Hymel, 1997; Nansel et al., 2001, 2004; Card and Hodges, 2010).

In fact, and in line with our results, previous research has demonstrated that victims of peer maltreatment feel lonelier, less happy, and have fewer good friends at school than their peers (Boulton and Underwood, 1992; Nansel et al., 2001, 2004). Furthermore, the lack of proper opportunities for anxiously withdrawn victimized adolescents to interact within the peer group and develop significant relationships at school (Haynie et al., 2001; Schwartz et al., 2001; Veenstra et al., 2005; Leary et al., 2006; Correia et al., 2014b) may be linked to heightened feelings of loneliness. It is important to note, however, that these feelings of loneliness did not extend to the family environment. It is possible that youths in the current study benefited from a strong family environment, characterized by a positive emotional bond among family members and feelings of closeness, which may have resulted in feelings of belonging and acceptance within the family system (Mckeown et al., 1997).

In sum, anxiously withdrawn victims were viewed by peers as more excluded, less aggressive, less prosocial, and less popular than non-withdrawn victims and non-victims. Non-anxiously withdrawn victims were considered more excluded than non-victims, and more aggressive than both anxiously withdrawn victims and non-victims. Finally, anxiously withdrawn victims reported feeling less integrated and intimate with their peers than non-withdrawn victims and non-victims, which is indicative of greater feelings of social and emotional loneliness at school. Youths in the current study did not report feeling lonely in their family environment. However, the possible positive relationship with their families did not seem to prevent victimization at school for either withdrawn or non-withdrawn adolescents.

The present study has several strengths to be noted. To our knowledge, this is the first study to examine the social behavior and perceived social and emotional loneliness across distinct subtypes of victimized youths in a Southern European country. From a methodological standpoint, the use of a multidimensional peer nomination measure (positive and negative dimensions of social behavior) and the use of a self-report measure that focuses on different aspects of loneliness across two major developmental contexts (family and peer group) both constitute an advance in the current state-of-the-art knowledge on the topic.

Some limitations must also be acknowledged. Participants were recruited using a convenience sampling method in two schools in Metropolitan Lisbon, limiting the ability to generalize the findings. The cross-sectional design of this study does not allow us to establish causal relationships between the variables examined, thus findings should be interpreted with caution. It would be important to further investigate the present findings, using longitudinal designs and a multimethod and multi-informant approach to validate peer nominations of social behavior (e.g., teacher reports or self-reports). Moreover, we recognize that, although we included an indirect measure of loneliness to prevent social desirability, the use of a self-report measure may have still influenced the results. Finally, the identification of risk groups and the procedure we used to assign adolescents to such groups may be considered a limitation. Some researchers have defended that a more data-driven strategy would be advisable and that the use of dimensions (i.e., continuous data) to classify children into groups, types, or categories results in the loss of information and of statistical power. However, it is also the case that a large body of research on peer relations, which uses peer assessment and sociometric procedures (frequently skewed or zero-inflated), rely on the classification method. Many of such studies have demonstrated that important and consistent behavioral differences exist between peers in extreme groups and their average, normative counterparts. These characteristics make them more likely than others to show an elevated level of maladjustment. The issue of continuous scores or categories has been debated within psychology about dimensions vs. types, namely about attachment, parenting practices, personality, among other things.

Our findings represent one more step along the route to helping professionals develop more effective prevention and intervention programs tailored to distinct subgroups of victimized adolescents. Particularly, governments and schools should encourage and invest in programs that focus on building resilience and improving anxious withdrawn youths' social skills and assertiveness in order to minimize their vulnerability to peer victimization and exclusion. Also, non-withdrawn victims may benefit from psychosocial programs that focus at promoting emotional and anger regulation.

## Data Availability Statement

The raw data supporting the conclusions of this article will be made available by the authors, without undue reservation.

## Ethics Statement

The studies involving human participants were reviewed and approved by ISPA-Instituto Universitário's Ethical Committee. Written informed consent to participate in this study was provided by the participants' legal guardian/next of kin.

## Author Contributions

AJS and KR: conception of the work. OR and MF: data collection. TA, MF, and OR: data analysis and drafting the manuscript. AJS, KR, OR, MF, and TA: data interpretation and edit the manuscript. All authors read and commented on the manuscript.

## Conflict of Interest

The authors declare that the research was conducted in the absence of any commercial or financial relationships that could be construed as a potential conflict of interest.
